# Inhibitory Function of the Dorsomedial Hypothalamic Nucleus on the Hypothalamic–Pituitary–Adrenal Axis Response to an Emotional Stressor but not Immune Challenge

**DOI:** 10.1111/j.1365-2826.2012.02369.x

**Published:** 2012-12-21

**Authors:** K Ebner, P Muigg, N Singewald

**Affiliations:** Department of Pharmacology and Toxicology, Institute of Pharmacy and Center for Molecular Biosciences Innsbruck (CMBI), Leopold-Franzens-University of InnsbruckInnsbruck, Austria

**Keywords:** dorsomedial hypothalamic nucleus, hypothalamic-pituitary-adrenal axis, adrenocorticotrophic hormone, paraventricular nucleus, lesion

## Abstract

Accumulating evidence implicates the dorsomedial hypothalamic nucleus (DMH) in the regulation of autonomic and neuroendocrine stress responses. However, although projections from the DMH to the paraventricular hypothalamic nucleus (PVN), which is the critical site of the neuroendocrine stress axis, have been described, the impact of DMH neurones in the modulation of hypothalamic-pituitary-adrenal (HPA) axis activation during stress is not fully understood. The present study aimed to investigate the role of the DMH in HPA axis responses to different types of stimuli. Male Sprague–Dawley rats fitted with a chronic jugular venous catheter were exposed to either an emotional stressor (elevated platform-exposure) or immune challenge (systemic interleukin-1β administration). Bilateral electrolytic lesions of the DMH disinhibited HPA axis responses to the emotional stressor, as indicated by higher plasma adrenocorticotrophic hormone levels during and after elevated platform exposure in lesioned animals compared to sham-lesioned controls. Moreover, DMH-lesioned animals showed increased neuronal activation in the PVN, as indicated by a higher c-Fos expression after elevated-platform exposure compared to controls. By contrast, DMH-lesions had no effects on HPA axis responses to immune challenge. Taken together, our data suggest an inhibitory role of DMH neurones on stress-induced HPA axis activation that is dependent upon the nature of the stimulus being important in response to an emotional stressor but not to immune challenge.

The dorsomedial hypothalamic nucleus (DMH) has been identified as a structurally and functionally diverse integrative structure involved in the regulation of physiological and behavioural stress responses 1–6. Evidence for the involvement of this region in stress regulation comes from functional immediate early gene expression studies demonstrating a substantial up-regulation of genes and/or proteins those for c-Fos in the DMH after exposure to various aversive and stressful stimuli 7–15. Moreover, there is considerable evidence that this area plays a key role in the regulation of stress-related cardiovascular and neuroendocrine functions 1–3,16. The latter functions are mediated by a discrete set of hypophysiotrophic neurones in the hypothalamic paraventricular nucleus (PVN), which initiate the hypothalamic-pituitary-adrenal (HPA) cascade by stimulating the release of adrenocorticotrophic hormone (ACTH), which in turn activates the secretion of glucocorticoids such as corticosterone from the adrenal glands 17,18. However, the exact role of the DMH in HPA axis regulation is not fully understood as a result of inconsistent findings in previous studies. For example, chemical stimulation of this region by the microinjection of drugs that block GABA_A_ receptor-mediated inhibition or stimulate ionotrophic glutamate receptors increases basal ACTH and/or corticosterone plasma concentrations in rats 19–21. Conversely, local microinjection of muscimol, a selective GABA_A_ receptor agonist, attenuates stress-induced elevations of ACTH levels 1996 and c-Fos expression in the PVN 23. Thus, these observations support a model that GABA acts locally within the DMH to inhibit excitatory output pathways mediating neuroendocrine stress responses 2. On the other hand, there is also evidence for an inhibitory influence of DMH neurones on HPA stress responses. For example, previous studies have shown that glutamatergic stimulation of DMH neurones generates GABAergic inhibitory post-synaptic potentials in PVN neurosecretory neurones 24, whereas blockade of glutamate receptors in the DMH by microinjection of kynurenic acid disinhibits stress-induced corticosterone levels 25. Moreover, combined tract tracing studies with c-Fos analysis indicate that a substantial number of stress-activated DMH neurones project to the PVN 9,26,27. Notably, the vast majority of stress-activated DMH neurones (almost 90%) were also found to co-express the GABAergic marker glutamic acid decarboxylase 26. Thus, these data point to a possible inhibitory role of the DMH on HPA axis activity. However, although GABAergic afferents to the PVN from the DMH have been described 28, direct functional evidence for a stress-activated GABAergic pathway from the DMH to the medial parvocellular PVN is still missing. Alternative attempts using lesioning techniques to determine the effects of ablation of this structure on HPA axis regulation are rare. The only two studies examining HPA axis activity following DMH lesions reveal no clear-cut picture because they were performed either only under basal conditions 29 or using specific challenging situations such as osmotic stress 30. However, none of these studies have examined the effects of DMH lesions on HPA axis function during emotional stressors.

The present study aimed to investigate the role of the DMH on basal and stress-induced HPA axis activity by studying the effects of surgical lesions of the whole DMH on HPA axis responses to different types of stimuli. Thus, we compared the basal and stimulated HPA axis activity of DMH-lesioned and sham-lesioned animals at different levels: (i) the hypothalamus, by quantifying the number of stress-induced c-Fos positive cells in the PVN as marker of neuronal activity and (ii) the pituitary, by measuring plasma ACTH levels. The exposure of animals to an elevated platform was used as an emotional stressor because this kind of stimulus has only minor physical components. As a non-emotional stimulus that potentially activates the HPA axis 31,32 we used an immune challenge induced by a single systemic interleukin (IL)-1β administration.

## Materials and Methods

### Animals

All experiments were carried out on adult male Sprague–Dawley rats (weighing 300–350 g). Before use, the animals were housed in groups of four to six under a 12 : 12 h light/dark cycle (lights on 07.00 h) at 21 ± 1 °C and 60% humidity, with pelleted food and water available *ad lib*., for at least 1 week after delivery from the supplier. All experiments were approved by the local Ethical Committee on Animal Care and Use of the Austrian governmental body.

### Surgery

All surgeries were conducted under sodium pentobarbital (40 mg/kg, i.p.) and ketamine (50 mg/kg, i.p.) anaesthesia. Moreover, before incision, the local anaesthetic xylocaine (AstraZeneca, Vienna, Austria) was injected on the head surface of animals. Postoperatively, rats were treated with buprenorphine (0.1 mg/kg body weight, s.c. every 8 h) and housed individually in transparent plexiglas cages until testing. They were handled for 3 min twice daily to familiarise them with the experimental procedure and to minimise nonspecific stress responses during the experiments. At least 16 h before experiments, animals were placed in the experimental room and allowed to habituate.

### DMH lesions

Bilateral lesions of the DMH were produced by passing a 1.5 mA anodal current for 9 s through a monopolar stainless steel electrode with a diameter of 0.2 mm according to the coordinates of a stereotaxic atlas 33 (coordinates: 2.9 mm caudal to bregma, 0.5 mm lateral to the midline, 8.8 mm below the surface of the skull). Sham-lesioned animals received the same treatment, except that the probe was only lowered 1 mm ventral to skull surface and no current was passed.

### Implantation of a jugular venous catheter

A silastic-tipped vinyl catheter was inserted into the left jugular vein, routed under the skin and exteriorised at the neck of the animal as described previously 34. The wounds were closed using metal clips. The catheter was filled with sterile saline containing gentamycin (30 000 IU/rat; Centravet, Bad Bentheim, Germany) and flushed with the same solution 2 days after surgery. On the day of the experiment, the catheters were connected to 1-ml plastic syringes via approximately 40-cm long pieces of PE-50 tubing 2 h before starting the experiment. Blood sampling through a pre-implanted jugular venous catheter allows repeated blood sampling from conscious, freely-moving rats without restraining animals.

### Experimental protocol

Three days after surgery, animals were separated into two groups, consisting of both sham- and DMH-lesioned animals. One group of animals (n = 18) was exposed to an elevated platform (circular plastic platform with a diameter of 24 cm elevated 70 cm above the floor) for 5 min, whereas the other group (n = 10) received a systemic administration of human recombinant IL-1β (1 μg/kg in 0.2 ml saline, i.v.). The experiment started with the collection of a first blood sample (0.3 ml) under basal conditions, 30 min before stress exposure. To determine the time course of plasma ACTH release in response to different stressors, additional blood samples were collected 10, 30 and 60 min after onset of the stressor. Sampled blood volumes were immediately replaced with an equal volume of heparinised saline. Blood samples were collected on ice, centrifuged and the plasma removed and stored at −20 °C for subsequent analysis of plasma ACTH levels by radioimmunoassay. All experiments were performed between 07.00 and 16.00 h. Exposure to the stressor was always completed between 11.00 h and 14.00 h to minimise circadian rhythm-related variations in stress responses.

### Immunocytochemistry

Two hours after stress exposure, lesioned and sham-lesioned animals were deeply anaesthetised with an overdose of sodium pentobarbital (200 mg/kg) and transcardially perfused with 100 ml of 0.9% saline followed by 100 ml of 4% paraformaldehyde in 0.1 m/l phosphate-buffered saline (PBS; pH 7.4). Brains were then removed, postfixed for 2 h, and cryoprotected at 4 °C overnight in 10% sucrose (in 0.1 m phosphate buffered saline, pH 7.4). Coronal sections (40 μm) were cut through the PVN using a Cryostat (Leica CM 1850; Leica-Microsystems, Nussloch, Germany) and collected in PBS. The sections were processed for c-Fos immunoreactivity as described previously 35. Briefly, brain sections were incubated for 48 h in rabbit anti-Fos (dilution 1 : 50 000; Santa Cruz Biotechnology, Santa Cruz, CA, USA), followed by a 2-h incubation in a biotinylated donkey anti-rabbit (dilution 1 : 300; Jackson ImmunoResearch, West Grove, PA, USA). Sections were then incubated for a further 2 h in a solution of avidin–biotin–horseradish peroxidase complex (ABC Vector Elite Kit; Vector Laboratories, Burlingame, CA, USA) before being exposed to a nickel 3,3′-diaminobenzidine solution to allow visualisation of horseradish peroxidase activity. Cells containing a nuclear brown–black reaction product were considered as c-Fos positive cells. The reaction was terminated once an optimal contrast between specific cellular and nonspecific background labelling was reached. Sections were then mounted on chrome-alum subbed slides, dehydrated in alcohol, cleared in xylene and cover-slipped.

### Quantification of c-Fos positive cells

Counts of c-Fos positive cells were made visually by an experimenter who was blind to experimental conditions. The total number of c-Fos-positive cells of the medial parvocellular PVN were quantified bilaterally over two rostrocaudal sections corresponding to the antero-medial and postero-lateral subdivisions of the PVN defined according to the stereotaxic boundaries outlined in the rat brain atlas 33. Cell counts were expressed as the number of positive nuclei per defined unit area (0.01 mm^2^). The results of all counts were expressed as the mean ± SEM.

### Quantification of plasma ACTH

Plasma ACTH concentrations were measured by radioimmunoassay using a commercially available kit (MP Biomedicals, Orangeburg, NY, USA) with an intra- and inter-assay variability of less than 10% and a lower limit of detection of 6 pg/ml. Cross-reactivities with other peptides derived from the pro-opiomelanocortin precursor protein such as β-endorphin, α-melanocyte-stimulating hormone or β-lipoprotein were < 0.9%.

### Histology

At the end of the experiments, animals were killed by an overdose of pentobarbital and their brains were removed. For histological verification of the extension of DMH lesions (Fig. 1), brains were sectioned using a cryostat and 40-μm coronal sections were stained with cresyl-violet. Lesion verification was performed before the analysing experiments.

**Figure:**
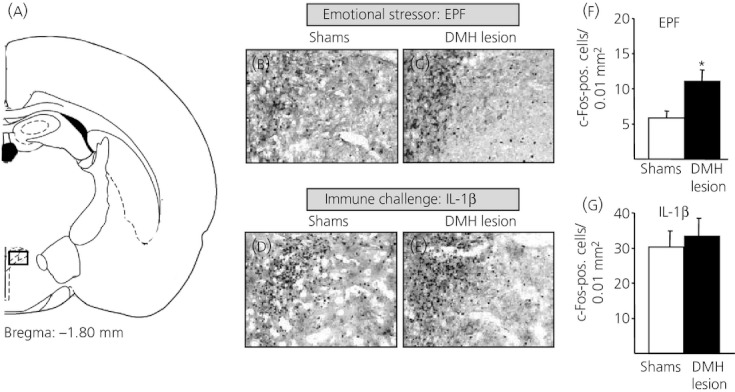
Coronal sections through the dorsomedial hypothalamic nucleus (DMH) to visualise the placement and extent of electrolytic lesions. Photomicrographs of representative complete DMH lesion (upper panels) and schematic illustrations of a rat brain (lower panels) adapted from a stereotaxic atlas 33 demonstrating the extent of lesions in the DMH are presented. The largest (bright grey) and smallest (dark grey) lesions are plotted at four different levels through the DMH in frontal sections. Other DMH lesions were intermediate in size and located in the same approximate area. 3V, third ventricle; f, fornix; ot, optical tract; VMH, ventromedial hypothalamic nucleus.

### Statistical analysis

Experimental subjects were included in the statistical analysis only if the lesions were confirmed to be localised within the DMH. Statistical analysis was performed using gb-stat, version 6.0 (Dynamic Microsystems, Silver Springs, MD, USA). Plasma ACTH concentrations were analysed using two-way anova (lesion × time) with repeated measures on the last factor followed by the appropriate post-hoc analysis. Counts of c-Fos-positive nuclei within the PVN after stress exposure were analysed using Student's two-tailed t-test comparing DMH-lesioned animals with sham-lesioned controls. Data are presented as the mean ± SEM. P < 0.05 was considered statistically significant.

## Results

### Lesion effects on neuroendocrine stress response

The placement and extent of each DMH lesion was verified on the basis of tissue destruction visualised in cresyl violet-stained sections (Fig.). The location of the DMH region was determined on the basis of previous definitions 33–36. However, animals with incomplete lesions or misplaced lesions were excluded from the analysis. Special care was taken not damage neighbouring areas, including the PVN or areas surrounding the PVN.

The effects of DMH lesions on ACTH levels were assessed before and after stress exposure. Under basal conditions, our results show no difference in ACTH levels of lesioned and sham-lesioned control rats (sham lesions: 42.2 ± 4.2; DMH lesions: 49.9 ± 5.8 pg/ml). As shown in Fig. 2(a), exposure to an elevated platform caused an increase in plasma ACTH levels in both DMH-lesioned animals and sham-lesioned controls. Statistical analysis of ACTH levels by two-way anova revealed a significant effect of the main factors (lesion: F_1,16_ = 4.53, P = 0.0492; time: F_3,48_ = 47.03, P < 0.0001). Post-hoc analysis indicated significant differences of ACTH levels between lesioned animals and controls during and immediately after stress exposure, with a higher ACTH response to elevated platform exposure at 10 (P < 0.05) and 30 min (P < 0.05) after onset of the stressor in lesioned animals compared to sham-lesioned controls.

**Figure:**
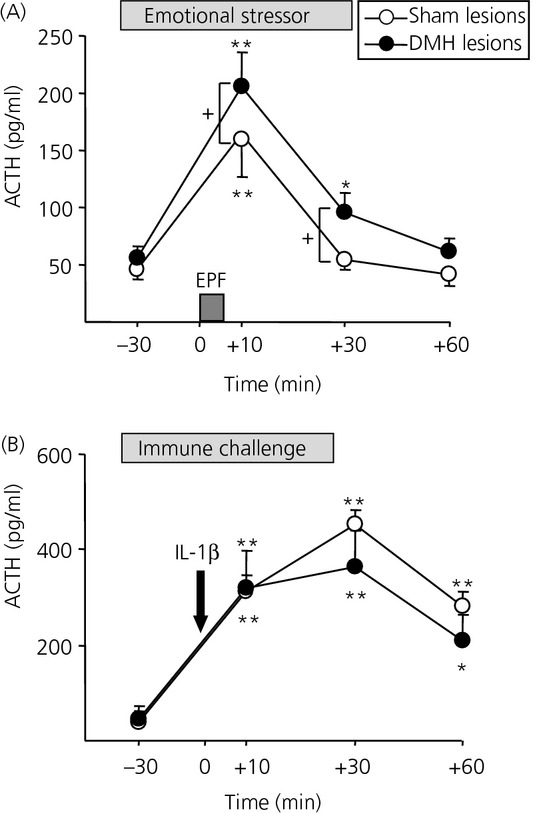
Effect of dorsomedial hypothalamic nucleus (DMH) lesions on adrenocorticotrophic hormone (ACTH) responses to (a) an emotional stressor (5-min elevated platform exposure, EPF; grey bar) and (b) immune challenge [systemic interleukin (IL)-1β administration, 1 μg/kg; arrow]. Both stimuli caused a significant increase in plasma ACTH concentrations in both DMH-lesioned and sham-lesioned animals. Although DMH-lesioned animals displayed higher plasma ACTH levels after EPF exposure compared to sham-lesioned animals, DMH lesions had no significant effect on plasma ACTH concentrations after IL-1β administration. *P < 0.05, **P < 0.01 versus basal; ^+^P < 0.05 versus respective value in the sham-lesioned control animals (two-way anova followed by Fisher's least significant difference post-hoc test).

Similar to elevated platform exposure, IL-1β administration resulted in significant increases in plasma ACTH concentrations in both lesioned and sham-lesioned animals (Fig. 2b). Notably, the stress-induced enhancement of ACTH was greater after IL-1β administration than after elevated platform exposure. In sham-lesioned animals, administration of IL-1β resulted in a higher and prolonged ACTH response, peaking at 30 min (450 ± 32 pg/ml), compared to an exposure to elevated platform (165 ± 22 pg/ml), which peaked immediately after stress exposure at 10 min and declined to basal levels by 30 min. However, in contrast to animals exposed to an elevated platform, lesions of the DMH did not affect ACTH concentrations in these animals (lesion: F = 0.53, P = 0.486; time: F = 33.22, P < 0.0001) because at no time point did ACTH levels differ between DMH-lesioned animals and sham-lesioned controls (Fig.b).

### Lesion effects on stress-induced PVN activation

Because we established that DMH lesions alter ACTH responses to elevated platform exposure but not to IL-1β, we next examined whether these different ACTH responses are associated by different neuronal activation levels in the PVN, the apex of HPA axis. Therefore, we determined the effects of DMH lesions on the expression of the c-Fos protein, a marker of neuronal activation, in the PVN of animals exposed to either elevated platform or systemic IL-1β administration. In previous studies, we have determined basal c-Fos expression in the PVN of rats as being very low (0.1–0.7 c-Fos-positive cells/0.01 mm) 37.

**Fig 3:**
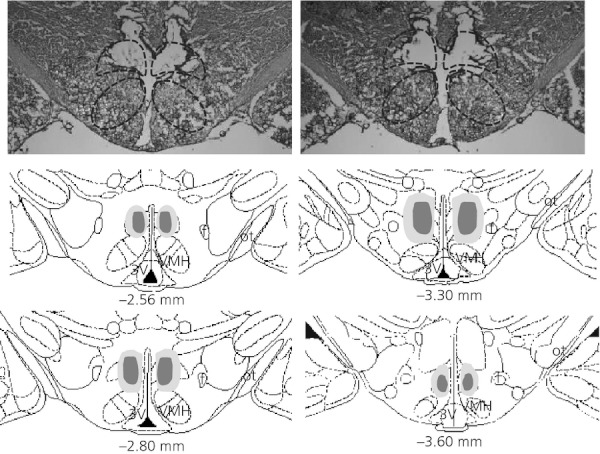
Effect of dorsomedial hypothalamic nucleus (DMH) lesions on the level of neuronal activation seen in the paraventricular nucleus (PVN) after elevated platform exposure (EPF) and systemic interleukin (IL)-1β administration. (a) Schematic diagram illustrating the location of the PVN. Rectangle indicates area of the PVN corresponding to photomicrographs. (b–e) Representative photomicrographs illustrating c-Fos expression in the medial parvocellular PVN. While DMH-lesioned animals (c) had more c-Fos-positive cells within the PVN after EPF exposure compared to sham-lesioned animals (b,f), DMH lesions had no effect on the c-Fos response to IL-1β administration (d,e,g). *P < 0.05 versus sham-lesioned controls (Student's t-test).

As shown in Fig. 3, sham-lesioned animals subjected to both stressors displayed high numbers of c-Fos-positive cells in the PVN, especially in the medial parvocellular part of the PVN. Notably, stress-induced c-Fos expression in the PVN was more pronounced after immune challenge than after platform exposure. Moreover, we found that DMH-lesioned animals exposed to elevated platform displayed more c-Fos-positive cells within the PVN than sham-lesioned controls. By contrast, DMH lesions had no effect on the c-Fos response to IL-1β administration as the number of c-Fos cells within the PVN did not differ between lesioned animals and sham-lesioned controls (Fig. 3).

## Discussion

The results of the present study demonstrate the substantial role of the DMH in the regulation of the HPA axis stress response. Our findings suggest that DMH neurones exert an inhibitory effect on stress-induced HPA axis activation as indicated by disinhibited plasma ACTH levels and exaggerated c-Fos expression in the PVN in DMH-lesioned animals compared to sham-lesioned controls. Interestingly, this effect appears to be dependent upon the nature of the stimulus, being important in response to an emotional stressor but not to immune challenge. Moreover, prestress (basal) ACTH levels did not differ in lesioned and control animals, indicating that the regulation of basal HPA axis activity is independent of the influence of the DMH.

### Basal HPA axis activity

Our findings suggest that DMH neurones are not involved in basal HPA axis regulation but are important in mediating HPA axis stress responses. Thus, our data are in contrast to previous findings demonstrating increased basal stress hormone levels after stimulation of DMH neurones via local microinjections of glutamate agonists or GABA antagonists 19–21 suggesting a tonic facilitatory role of DMH neurones on HPA axis activity. However, this discrepancy may be related to methodological differences (e.g. type and site of intervention, time scale, blood collection procedure). Moreover, the fact that most of these previous pharmacological studies investigated the role of a distinct population of neurones (e.g. GABAergic neurones) within the DMH might also be relevant. In this context, it should be noted that the role of the DMH in HPA axis regulation may be subregion or cell-type specific. Indeed, electrophysiological data indicate that this region supplies excitatory as well as inhibitory input to PVN neurones 24,28. Thus, the effects that are finally produced depend on the exact position of the manipulation or ablation of DMH neurones. The only two studies investigating the effects of surgical elimination of the whole DMH on HPA axis function revealed either higher corticosterone levels 29,30 or a disruption of the normal diurnal plasma corticosterone rhythm in rats with bilateral DMH lesions 29. However, the results of the latter study may have been influenced by technical issues because stress hormone measurements were taken from heart puncture. Blood sampling under light ether anaesthesia, as performed for heart puncture in the study by Bellinger *et al*. (29), is problematic because most anaesthetics increase HPA axis activity 39,40. It is not surprising that stress hormone levels measured in the previous study are higher than normal resting levels in male rats during the morning hours 41. Thus, our data showing unchanged basal stress hormone levels appear to be more physiological because we took blood samples from conscious, freely-moving animals via an i.v. implanted catheter without any further stressful or anaesthetic procedures.

### Stress-induced HPA axis activity

The present study demonstrates that lesions of the DMH increase HPA axis stress responses. These findings are consistent with the idea that an intact DMH inhibits neuroendocrine stress responses and support previous findings demonstrating an inhibitory input of DMH neurones to PVN neurosecretory neurones (see Introduction). Moreover, these data confirm previous findings reporting increased stress-induced corticotrophin-releasing hormone mRNA levels in the PVN, as well as increased anterior pituitary pro-opiomelanocortin mRNA levels in animals after surgical lesion of the central part of the DMH 30. Notably, in this previous study, i.p. hypertonic saline injection was used as an osmotic stressor, which is known as an aversive stimulus with painful and psychological stress components 42. On the other hand, findings of DiMicco's group demonstrate the opposite effect an attenuation of HPA axis responses to air jet stress after inactivation of the DMH via muscimol microinjections, suggesting an excitatory rather than inhibitory influence of DMH on HPA axis activity (see Introduction). The apparent discrepancy between these results and those of the present study may be a result of technical and methodological differences. As already mentioned, these former studies used a pharmacological approach to temporarily inactivate the DMH by microinfusions of the GABA_A_ receptor agonist muscimol, whereas the present study was carried out using a permanent surgical lesion technique. Although each method has its advantages and limitations, one common disadvantage of chemical inactivation procedures such as muscimol infusion is a considerable uncertainty about the size and shape of the volume of the drug-infused tissue. Thus, it is possible that infusions of muscimol into the DMH spread to neighbouring structures involved in HPA axis activation. Moreover, chemical inactivating techniques can be specifically selective for distinct populations of neurones that make general conclusions difficult to draw. In respect of this, anatomically segregated neuronal populations that activate or inhibit HPA axis activity within the DMH 5,43 might further complicate things. An additional factor that could explain some of the discrepancy between results obtained in the present study and the findings of DiMicco's group 2,22,23 might be the modality of used stressors, with a mild elevated platform test with some locomotor components being used in the present study versus a strong immobilisation stress in which movements are restricted being used by DiMicco's group. However, our findings of an exaggerated/disinhibited neuroendocrine stress response after ablation of the complete DMH indicate an inhibitory net effect of this area on HPA axis activity during stress exposure. It is possible that some of the results found in the present study are a result of the destruction of fibres of passage through the DMH in addition to damage of the DMH itself. Further studies using excitotoxic lesion agents (e.g. ibotenic acid) that spare fibres of passage should address this issue to exclude the contribution of structures projecting through the DMH to the PVN.

Notably, we found that lesions of the DMH were associated with an increased plasma ACTH response to an emotional stressor such as elevated platform exposure but not to systemic IL-1β administration. This stimulus-dependent effect was paralleled by an exaggerated c-Fos expression in the PVN of DMH-lesioned animals exposed to an elevated platform. By contrast, cellular activation in the PVN after immune challenge was similar in DMH-lesioned rats and sham-lesioned controls. Although several explanations might account for this observation, a ceiling effect in response to immune challenge can be excluded because other stressors such as immobilisation stress elicited a more pronounced ACTH response than IL-1β administration (K. Ebner, unpublished observations) 41. Although it is not possible to rule out the possibility that cytokine production during recovery from surgery could mask the effect of IL-1β injection, this is unlikely. First, basal and stress-induced HPA axis activity (e.g. ACTH levels; Fig.) in animals with brain surgery and/or jugular vein catheterisation 24–72 h before sampling is in a range similar to that in non-operated animals 44,45. Second, our telemetry data show that brain surgery and venous catheterisation have no effect on blood pressure and heart rate measurements at 72 h after surgery (N. Singewald, unpublished observations). Third, after 72 h of recovery animals show no behavioural abnormalities with respect to exploration/locomotor activity or emotional-related behaviour as tested in the elevated plus maze test (K. Ebner, unpublished observations) 44. The most reasonable explanation is that DMH neurones are not involved in the regulation of HPA axis responses to a systemic stimulus such as immune challenge. Support for such a stimulus specific regulation comes from neuroanatomical studies demonstrating increased c-Fos expression in the DMH after emotional stressors such as forced swim, restraint, open-field, air-puff or foot-shock 8,10,11,15 but not after systemic stressors such as haemorrhage or ether inhalation 11,15 suggesting that the DMH is activated in response to the former but not in response to the latter stimulus type. Further support for the hypothesis that the DMH mediates HPA responses to emotional stressors comes from neuroanatomical studies combining retrograde tracing with c-Fos expression. These studies identified a large population of PVN projecting neurones in the DMH that are activated by emotional stressors such as swim stress or foot-shock 9,27 suggesting that these cells are involved in the regulation of HPA responses to these stressors. By contrast, systemic stimuli such as immune challenge appear to be mediated through innervations of the PVN from medullary autonomic sites 46. Along these lines, transection of ascending projections from the brain stem prevents c-Fos expression in the PVN in response to immune challenge but not to foot shock 47, suggesting a parallel dichotomy for the mechanisms involved in these two modes of stress. Thus, although both an elevated platform exposure and immune challenge provoked an extensive activation of neurones in the PVN, different afferent pathways appear to mediate the effects of each stressor.

In summary, our data demonstrate that ablation of the DMH, an area known to send projections to the parvocellular PVN 9,48,49 results in a disinhibition of HPA axis stress responses that is dependent upon the nature of the stimulus used. Thus, the results of the present study confirm and extend previous work suggesting an inhibitory role of DMH neurones on neuroendocrine stress responses. Considering this evidence of a stress-inhibitory action of the DMH, it might be speculated that dysfunctions in this area underlie the inadequate responding to (aversive) emotional challenges, resulting in exaggerated and/or prolonged stress responses, which are typical signs in a number of stress-related psychopathologies.
